# Medicaid Payments and Racial and Ethnic Disparities in Alzheimer Disease Special Care Units

**DOI:** 10.1001/jamanetworkopen.2025.25057

**Published:** 2025-08-04

**Authors:** Huiwen Xu, Shuang Li, John R. Bowblis, Monique R. Pappadis, Yong-Fang Kuo, James S. Goodwin

**Affiliations:** 1 Nell Hodgson Woodruff School of Nursing, Emory University, Atlanta, Georgia; 2 Sealy Center on Aging, University of Texas Medical Branch, Galveston; 3 Department of Economics, Farmer School of Business, Miami University, Oxford, Ohio; 4 Scripps Gerontology Center, Miami University, Oxford, Ohio; 5 School of Public and Population Health, University of Texas Medical Branch, Galveston; 6 Department of Internal Medicine, University of Texas Medical Branch, Galveston

## Abstract

**Importance:**

Alzheimer disease special care units (ASCUs) are associated with improved outcomes for residents with dementia, yet they are unavailable in most nursing homes.

**Objectives:**

To examine racial and ethnic disparities in the availability of ASCUs and whether more generous Medicaid payments are associated with reduced disparities.

**Design, Setting, and Participants:**

This cohort study used 2009-2019 Certification and Survey Provider Enhanced Reporting data and resident assessments from Medicare- and Medicaid-certified nursing homes in the US, as well as state Medicaid payment-to-cost ratios for 2019. Statistical analysis was performed from September to December 2024.

**Exposure:**

The percentages of Black residents and Hispanic residents in a facility and the state mean nursing homes’ ratio of Medicaid payment to estimated Medicaid cost of care.

**Main Outcomes and Measures:**

The main outcome was whether a nursing home had an ASCU. Multivariable logistic regression was conducted on ASCUs, and then separate logistic regressions were performed for states with different quartiles of Medicaid payment-to-cost ratios.

**Results:**

Most of the 13 229 nursing homes in the study were for profit (9561 [72.3%]) and were part of a chain (7775 [58.8%]). The overall mean (SD) Medicaid payment-to-cost ratio among all states was 0.87 (0.13) (range, 0.58-1.29). Each 1% increase in the percentage of Black residents was associated with a 0.1% decrease in the probability of having an ASCU. Compared with facilities with 0% to 0.8% of Black residents, the odds of having an ASCU were 37% lower in nursing homes with 4.3% to 15.2% Black residents (odds ratio [OR], 0.63; 95% CI, 0.53-0.74), and 45% lower in nursing homes with 15.2% or more of Black residents (OR, 0.55; 95% CI, 0.46-0.65). Compared with facilities with no Hispanic residents, the odds of having an ASCU were 27% lower in those with 3.7% or more of Hispanic residents (OR, 0.73; 95% CI, 0.62-0.86). In states with Medicaid payment-to-cost ratios between 0.58 and 0.81, nursing homes with 15.2% or more of Black residents were 68% less likely to have an ASCU (OR, 0.32; 95% CI, 0.21-0.50). This difference decreased to 45% in states with Medicaid payment-to-cost ratios between 0.82 and 0.94 (OR, 0.55; 95% CI, 0.44-0.69) and almost disappeared in states with Medicaid payment-to-cost ratios greater than 0.94 (OR, 0.86; 95% CI, 0.53-1.40). Higher Medicaid payment-to-cost ratios were not associated with reduced disparities among Hispanic residents.

**Conclusions and Relevance:**

This cohort study of nursing homes suggests that racial and ethnic disparities in ASCU availability narrowed in states where Medicaid payment rates cover a greater share of costs. Racial disparities in specialized dementia care may be mitigated and even eliminated by more generous Medicaid payments.

## Key Points

**Question:**

Are more generous Medicaid payments associated with a decrease in racial and ethnic disparities in the availability of Alzheimer disease special care units in nursing homes?

**Findings:**

In this cohort study of 13 229 nursing homes, those with higher proportions of Black or Hispanic residents were less likely to have Alzheimer disease special care units. The disparities among nursing homes serving high proportions of Black residents, however, narrowed and even disappeared in states with higher Medicaid payment-to-cost ratios.

**Meaning:**

This study suggests that more generous Medicaid payments may be associated with improved availability of specialized dementia care in nursing homes that serve primarily marginalized Black residents.

## Teaser

This cohort study examines racial and ethnic disparities in the availability of Alzheimer disease special care units in US nursing homes and whether more generous Medicaid payments are associated with reduced disparities.

## Introduction

Approximately 750 000 older adults with Alzheimer disease and related dementias reside in nursing homes.^1,2,3^ To meet their unique care needs, some nursing homes have developed specialized dementia care programs.^1,4,5,6^ These Alzheimer disease special care units (ASCUs) typically include a modified physical environment and staff trained to prevent or reduce the impact of adverse behaviors, such as wandering.^6,7,8^ Evidence suggests that ASCUs are associated with improved resident outcomes. Residents in facilities with an ASCU are less likely to be restrained, receive antipsychotic medications, develop pressure ulcers, or be hospitalized.^5,6,7,8^ Resident and family satisfaction is higher not only for those with dementia but also for those in the same facility without dementia and not in the ASCU.^6^ A recent study also found some positive spillover effect of ASCUs on residents of short-stay skilled nursing homes.^4^

However, access to the benefits associated with ASCUs may not be equally distributed. Racial and ethnic disparities in quality of care and health outcomes among nursing home residents, including those with dementia, have been well documented.^9,10,11,12,13,14,15,16,17,18,19,20,21^ Compared with White residents, Black residents are more often physically restrained,^14^ more frequently develop pressure ulcers,^18^ are more frequently hospitalized,^13^ and report lower quality of life.^16^ Hispanic residents experience a higher prevalence of pressure ulcers,^18^ worse symptom management,^19^ more avoidable hospitalizations,^20^ and worse end-of-life care.^21^

The worse outcomes experienced by Black and Hispanic residents may be partially due to lack of access to ASCUs. Despite their potential benefits for residents with dementia and their families, ASCUs are available in only about 15% of all US nursing homes,^22^ and the presence of ASCUs varies substantially across states.^22^ Medicaid has been identified as a key factor associated with lower-quality care and racial and ethnic disparities.^23^ Black and Hispanic US residents are more likely to rely on Medicaid (vs private insurance or out-of-pocket payments) to pay for nursing home care.^24^ Medicaid coverage represents a potential barrier to ASCU access. First, Medicaid payment rates are lower than private pay rates.^25^ Second, private pay rates vary by whether the resident is in an ASCU, but Medicaid typically pays a flat per diem with adjustment for facility average case mix.^25^

State Medicaid payment rates for nursing home care vary more than 2-fold.^26,27,28^ Nursing homes in states with higher Medicaid payment rates have higher staffing levels,^29^ lower hospitalization rates,^30,31^ fewer emergency department visits,^32^ and more discharges to the community.^27^ A major limitation of using Medicaid payment rates is that they ignore the cost of nursing home operations. Mean daily nursing home operating costs vary from less than $200 in Arkansas, Georgia, and Texas to more than $300 in New York, North Dakota, and Oregon.^26^ A ratio of Medicaid payment rates to costs can better capture the generosity of Medicaid payments.

In this study, we address 2 questions. First, are there racial and ethnic disparities in the availability of ASCUs? Second, if so, is it a function of the level of Medicaid reimbursement? We hypothesize that nursing homes with higher proportions of Black or Hispanic residents will be less likely to have ASCUs and that those disparities will be reduced in nursing homes in states with more generous Medicaid payments.

## Methods

### Data Sources

The 2009-2019 Certification and Survey Provider Enhanced Reporting (CASPER) data provide detailed nursing home characteristics, including the number of ASCU beds.^33^ CASPER data were collected as part of annual recertification inspections of all Medicare- and Medicaid-certified nursing homes.^34,35^ We did not use 2020 and 2021 data because the Centers for Medicare & Medicaid Services suspended recertification surveys early in the COVID-19 pandemic, and data in 2022 and beyond were unavailable for use. The 2009-2019 national Minimum Data Set (MDS) assessments collect resident race and ethnicity information.^36^ The Payroll-Based Journal staffing data were used to calculate total staff levels for 2019.^37^ The Medicaid and CHIP Payment and Access Commission (MACPAC) estimates the 2019 state daily Medicaid base payment rate and acuity-adjusted Medicaid costs from Medicaid claims and Medicare cost reports.^26^ MACPAC develops those estimates by facility and then aggregates to the state level.^26^ Finally, Medicaid reports calculate state Medicaid spending on home and community-based services as a percentage of total spending on long-term services and support.^38^ This study was approved by the Emory University institutional review board, with a waiver of informed consent because the data were deidentified. This study followed the Strengthening the Reporting of Observational Studies in Epidemiology (STROBE) reporting guideline.

### Study Population

The primary cohort included all Medicare- and Medicaid-certified nursing homes that had a 2019 CASPER survey in the 50 US states (N = 14 058; eFigure 1 in Supplement 1). The District of Columbia was excluded because of the lack of CASPER data. We excluded hospital-affiliated facilities that mostly focus on postacute care residents (n = 574) and facilities with missing racial and ethnic composition data (n = 17), and we restricted the sample to facilities from the 47 states with available Medicaid payment-to-cost ratios. Data from Alaska, Idaho, and New Hampshire were unavailable in the MACPAC report but were included in the descriptive analyses that did not require MACPAC data. Finally, we excluded 120 nursing homes where staffing levels were missing. In longitudinal analyses, we included 16 273 nursing homes with available CASPER data from 2009 to 2019.

### Measures

The main outcome was whether a nursing home had an ASCU.^22^ The primary exposure variables were the percentages of Black residents and Hispanic residents in a facility.^39^ The MDS collects self-reported race and ethnicity information of residents (eTable 1 in Supplement 1). The MDS that we used has only 1 question for race and ethnicity, so we cannot separate resident race from ethnicity. We calculated the percentages for all nursing home residents who had any MDS assessment in a facility for each year. In the multivariable analyses, we categorized nursing homes based on quartile of percentage of Black residents (Q1, 0%-0.78%; Q2, 0.79%-4.24%; Q3, 4.25%-15.16%; and Q4, ≥15.17%) and quartile of percentage of Hispanic residents (Q1, 0%; Q2, 0.01%-0.79%; Q3, 0.80%-3.73%; and Q4, ≥3.74%).^40^ The policy variable was the state Medicaid payment-to-cost ratio that measured the relative generosity of Medicaid base payments to nursing homes, with values greater than 1 indicating that Medicaid pays more than estimated costs.^26^ Because the Medicaid payment-to-cost ratio is a state-level variable, we first split the ratios into 4 quartiles by state based on its distribution. Our analyses found that states with ratios in Q2 and Q3 behaved similarly, justifying a categorization of 3 groups: Q1 (0.58-0.81), Q2 to Q3 (0.82-0.94), and Q4 (0.94-1.29), corresponding to Medicaid payments that were very constrained (Q1), moderately constrained (Q2-Q3), and sufficient (Q4).

We extracted the following facility covariates that might be associated with the availability of ASCUs (eTable 2 in Supplement 1): for-profit status (vs nonprofit or public status), chain affiliation, location, bed size, percentage of Medicaid residents, percentage of Medicare residents, percentage of residents with dementia, percentage of residents with depression, percentage of residents with serious mental illness, and total nursing staff hours per resident day.^22,35,41,42^ We obtained the percentage of state Medicaid spending on home- and community-based services because that may affect the population that nursing homes serve.^27,38^

### Statistical Analysis

Statistical analysis was performed from September to December 2024. We first presented the variation among the 50 states in the percentages of nursing homes that had ASCUs in each state. We described the characteristics of nursing homes in 2019 and also stratified by the quartile of percentage of Black residents. We plotted the percentage of each nursing home. We then grouped nursing homes with the same percentage of Black residents and calculated the percentage of facilities in each percentile with an ASCU. These data were then presented as a scatterplot. We conducted sensitivity analyses on 2009-2019 data to describe the time trend in the availability of ASCUs in a nursing home by their proportion of Black residents. We repeated the analyses for the percentage of Hispanic residents.

Using 2019 data, we conducted multivariable logistic regression of the 13 229 nursing homes in the 47 states with MACPAC data, with the dependent variable being the presence of an ASCU. In these analyses, percentages of Black residents and Hispanic residents were categorized by quartiles, using facilities in Q1 as the reference group. The multivariable model controlled for the facility characteristics described in the previous section. Robust SEs that accounted for the clustering of facilities within a state were used to calculate the 95% CI of the odds ratio (OR) for the percentages of Black residents and Hispanic residents. We also assessed racial and ethnic disparities in ASCUs using both percentages as continuous variables on 2009-2019 data.

To evaluate whether the generosity of Medicaid payment was associated with racial and ethnic disparities in ASCUs, we included the state Medicaid payment-to-cost ratios in the multivariable logistic regression as a facility characteristic. We then tested for an interaction between Medicaid payment-to-cost ratios and the percentage of Black residents. For interpretability, we conducted 3 separate multivariable logistic regressions in states with Medicaid payment-to-cost ratios in Q1, Q2 to Q3, and Q4. We repeated the analyses for the percentage of Hispanic residents in a facility and controlled for the percentage of state Medicaid spending on home- and community-based services. The analyses were performed using SAS, version 9.4 (SAS Institute Inc). All *P* values were from 2-sided tests and results were deemed statistically significant at *P* < .05.

## Results

**Figure 1.**
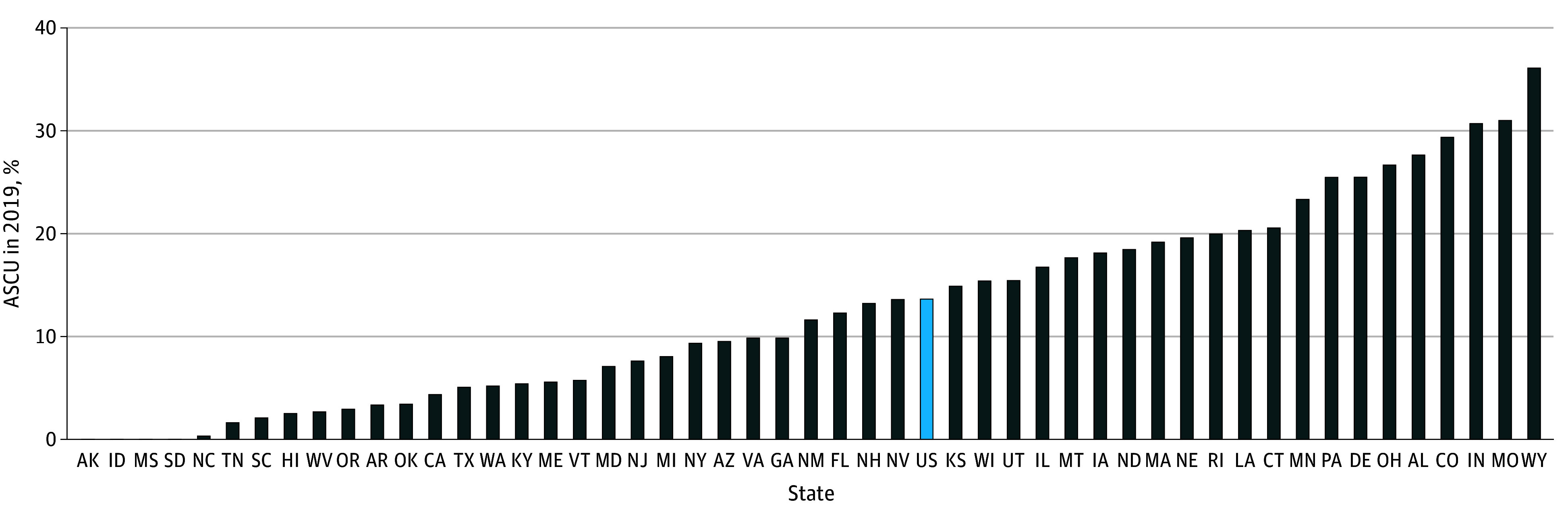
State Variation in the Availability of Alzheimer Disease Special Care Units (ASCUs) in Nursing Homes, 2019 The sample included 14 058 nursing homes in 50 states from the 2019 Certification and Survey Provider Enhanced Reporting. States were ranked based on the percentage of nursing homes with an ASCU.

Our primary cohort included 13 229 facilities in 2019 (86.3% of all certified nursing homes). Most facilities were for profit (9561 [72.3%]), part of a chain (7775 [58.8%]), and located in urban areas (9393 [71.0%]) (eTable 3 in Supplement 1). Facility characteristics differed by quartiles of percentages of Black residents (eTable 3 in Supplement 1) and Hispanic residents (eTable 4 in Supplement 1). The longitudinal cohort included 162 813 facility-year observations from 2009 to 2019 (range, 14 369-15 186 facilities per year). In 2019, ASCUs were available in 13.6% of all nursing homes in the US (1918 of 14 058) (Figure 1). There was no clear geographic pattern of states with high vs low percentages of nursing homes with ASCUs (eFigure 2 in Supplement 1). The prevalence was more than 30% in Alabama, Colorado, Indiana, Missouri, and Wyoming but 0% to 3% in Alaska, Idaho, Mississippi, South Dakota, North Carolina, Tennessee, South Carolina, Hawaii, West Virginia, and Oregon.

**Figure 2.**
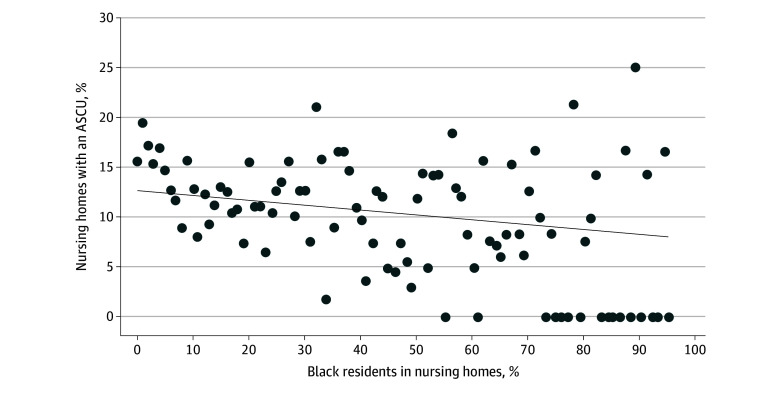
Association Between the Percentage of Black Residents in a Nursing Home and the Presence of an Alzheimer Disease Special Care Unit (ASCU), 2019 The sample included 13 229 nursing homes in 47 states from the 2019 Certification and Survey Provider Enhanced Reporting merged with Minimum Data Set 3.0 assessments. The percentage of Black residents was calculated as the number of Black residents in 2019 in a facility divided by the total number of residents. Nursing homes with the same percentage of Black residents were grouped together. The number of nursing homes grouped at each percentile from 0% to 60% are 20 or more. For percentiles greater than 60%, the number of facilities were usually less than 20. The dotted line indicates a simple linear regression between percentage of Black residents and ASCU. The slope of the regression line was −0.05% (*P* = .07).

There was an inverse association between the percentage of Black residents in a facility and ASCU availability (Figure 2). Each 1% increase in the percentage of Black residents was associated with a 0.1% decrease in the probability of having an ASCU. Similarly, the probability of having an ASCU was lower in nursing homes with a higher percentage of Hispanic residents (eFigure 3 in Supplement 1). We also examined the availability of ASCUs from 2009 to 2019 separately by the percentages of Black residents (eFigure 4 in Supplement 1) and Hispanic residents (eFigure 5 in Supplement 1) in a facility. Racial and ethnic disparities in the percentage of facilities with ASCUs were persistent over that period.

A multivariable logistic regression using 2019 data examined the association of the percentages of Black residents and Hispanic residents in a facility with the presence of an ASCU, controlling for facility characteristics described in the previous section (Table 1). Compared with facilities with 0% to 0.78% of Black residents, the odds of having an ASCU were not significantly different in nursing homes with 0.8% to 4.2% of Black residents (OR, 0.91; 95% CI, 0.79-1.05) but were 37% lower in facilities with 4.3% to 15.2% of Black residents (OR, 0.63; 95% CI, 0.53-0.74) and 45% lower in nursing homes with 15.2% or more of Black residents (OR, 0.55; 95% CI, 0.46-0.65). Compared with facilities without Hispanic residents, the odds of having an ASCU were not significantly different in nursing homes with 0.01% to 0.8% or 0.8% to 3.7% of Hispanic residents but were 27% lower in facilities with 3.7% or more of Hispanic residents (OR, 0.73; 95% CI, 0.62-0.86). Larger facilities, those that were nonprofit, part of a chain, or with lower percentages of short-stay (Medicare) residents and higher percentages of residents with dementia, depression, or serious mental illness were all more likely to have ASCUs. Results on 2009-2019 data were consistent when treating the percentages of Black residents and Hispanic residents as continuous variables (eTable 5 in Supplement 1).

**Table 1.** Multivariable Logistic Regression Examining Facility Characteristics Associated With Availability of Alzheimer Disease Special Care Unit Among 13 229 Nursing Homes, 2019

Facility characteristics	Odds ratio (95% CI)	
	Unadjusted^a^	Adjusted^b^
Percentage of Black residents^c^		
Q1 (0-0.78)	1 [Reference]	1 [Reference]
Q2 (0.79-4.24)	0.91 (0.80-1.04)	0.91 (0.79-1.05)
Q3 (4.25-15.16)	0.64 (0.55-0.73)	0.63 (0.53-0.74)
Q4 (≥15.17)	0.56 (0.48-0.64)	0.55 (0.46-0.65)
Percentage of Hispanic residents^c^		
Q1 (0)	1 [Reference]	1 [Reference]
Q2 (0.01-0.79)	1.37 (1.20-1.57)	1.45 (1.24-1.69)
Q3 (0.80-3.73)	1.03 (0.91-1.17)	1.14 (0.98-1.31)
Q4 (≥3.74)	0.69 (0.60-0.79)	0.73 (0.62-0.86)
Total staff hours per resident day		
Q1 (≤3.29)	1 [Reference]	1 [Reference]
Q2 (3.30-3.67)	1.10 (0.96-1.27)	1.09 (0.94-1.26)
Q3 (3.68-4.16)	1.16 (1.01-1.34)	1.22 (1.05-1.42)
Q4 (≥4.17)	0.98 (0.85-1.13)	1.09 (0.91-1.30)
Profit status		
For profit	1 [Reference]	1 [Reference]
Nonprofit or government	2.02 (1.82-2.23)	1.57 (1.39-1.78)
Part of chain		
No	1 [Reference]	1 [Reference]
Yes	1.11 (1.01-1.22)	1.49 (1.33-1.67)
Urban		
No	1 [Reference]	1 [Reference]
Yes	0.85 (0.76-0.94)	0.91 (0.80-1.03)
Bed size, per 10-bed increase	1.07 (1.06-1.08)	1.10 (1.09-1.11)
Percentage of Medicaid residents, per 10-unit increase	1.04 (1.02-1.06)	0.97 (0.95-1.01)
Percentage of Medicare residents, per 10-unit increase	0.65 (0.61-0.69)	0.72 (0.67-0.78)
Percentage of residents with dementia, per 10-unit increase	1.49 (1.45-1.54)	1.43 (1.38-1.48)
Percentage of residents with depression, per 10-unit increase	1.10 (1.07-1.12)	1.05 (1.02-1.07)
Percentage of residents with serious mental illness, per 10-unit increase	1.11 (1.08-1.14)	1.06 (1.02-1.09)

Abbreviation: Q, quartile.

^a^ Unadjusted odds ratios were obtained from separate simple logistic regressions.

^b^ Adjusted odds ratios were obtained from a multivariable logistic regression that included all the facility characteristics. Robust SEs were used.

^c^ The association was robust when treating the percentage of Black residents and the percentage of Hispanic residents as continuous variables.

Both Medicaid payment rates and the cost of providing care to Medicaid residents varied across states (eTable 6 and eFigure 6 in Supplement 1). The overall mean (SD) Medicaid payment-to-cost ratio among all states was 0.87 (0.13) (range, 0.58-1.29). We found a significant interaction between the percentage of Black residents and Medicaid payment-to-cost ratios on the availability of ASCUs (eTable 7 in Supplement 1). Table 2 presents 3 separate logistic regressions examining disparities in the availability of ASCUs in states with different Medicaid payment-to-cost ratios. In states with very constrained Medicaid payment-to-cost ratios (0.58-0.81), nursing homes with 0.8% to 4.2% of Black residents (Q2) had a similar likelihood of having an ASCU (OR, 0.83; 95% CI, 0.61-1.14), while nursing homes with 4.3% to 15.2% of Black residents (Q3) were 38% less likely to have an ASCU (OR, 0.62; 95% CI, 0.42-0.90) and nursing homes with 15.2% or more of Black residents (Q4) were 68% less likely to have an ASCU (OR, 0.32; 95% CI, 0.21-0.50) compared with facilities with 0% to 0.8% of Black residents (Q1). In states with moderately constrained Medicaid payment-to-cost ratios (0.82-0.94), nursing homes with higher proportions of Black residents were less likely to have an ASCU (Q3: OR, 0.58; 95% CI, 0.48-0.72; Q4: OR, 0.55; 95% CI, 0.44-0.69). In states with the highest Medicaid payment-to-cost ratios (0.94-1.29), the percentage of Black residents was no longer associated with ASCUs (Q4: OR, 0.86; 95% CI, 0.53-1.40). Results were consistent after controlling for the percentage of state Medicaid spending on home- and community-based services (eTable 8 in Supplement 1).

**Table 2.** Disparities in the Availability of Alzheimer Disease Special Care Unit by Percentage of Black Residents by State Medicaid Payment-to-Cost Ratios, 2019

Characteristic	Odds ratio (95% CI)^a^		
	Q1 (0.58-0.81)	Q2 to Q3 (0.82-0.94)	Q4 (0.94-1.29)
No. of nursing homes	3060	8589	1580
No. of states^b^	12	24	11
Percentage of Black residents			
Q1 (0-0.78)	1 [Reference]	1 [Reference]	1 [Reference]
Q2 (0.79-4.24)	0.83 (0.61-1.14)	0.86 (0.72-1.03)	1.31 (0.82-2.10)
Q3 (4.25-15.16)	0.62 (0.42-0.90)	0.58 (0.48-0.72)	0.82 (0.48-1.40)
Q4 (≥15.17)	0.32 (0.21-0.50)	0.55 (0.44-0.69)	0.86 (0.53-1.40)

Abbreviation: Q, quartile.

^a^ Three separate multivariable logistic regressions with robust SEs were conducted in states with Medicaid payment-to-cost ratios in Q1, Q2 to Q3, and Q4. The quartiles of Medicaid ratios were based on the number of states. Models controlled for the same facility characteristics as in Table 1. The interaction of the continuous percentage of Black residents with the continuous Medicaid payment-to-cost ratios was significant (*P* < .01). The findings are consistent with results from 1 logistic regression with the interaction term of the percentage of Black residents with Medicaid payment-to-cost ratios. The findings are robust after controlling for the state Medicaid spending on home- and community-based services. The interaction of the percentage of Hispanic residents with Medicaid payment-to-cost ratios was not significant (*P* = .37; eTable 9 in Supplement 1), and the reduced disparities were not observed in states with the highest Medicaid payment-to-cost ratios (eTable 10 in Supplement 1).

^b^ Nursing homes in 3 states and the District of Columbia were excluded from the analysis. Data from Alaska, Idaho, and New Hampshire were not available in the Medicaid and CHIP Payment and Access Commission report. The Certification and Survey Provider Enhanced Reporting data did not include nursing homes from the District of Columbia.

However, the interaction between the percentage of Hispanic residents and Medicaid payment-to-cost ratios on the availability of ASCUs was not significant (eTable 9 in Supplement 1). Also, reduced disparities in states with the highest Medicaid payment-to-cost ratios were not found for the percentage of Hispanic residents (eTable 10 in Supplement 1).

## Discussion

Using national nursing home data from 2009 to 2019, we found persistent racial and ethnic disparities in the availability of ASCUs. Previous studies have documented racial and ethnic disparities in nursing home care, including quality of life, end-of-life care, use of physical restraints, and development of pressure ulcers.^11,13,16,24^ Our findings point out a structural barrier—the relative lack of ASCUs—that may account for some of the prior findings on nursing home disparities.

Our results suggest that nursing homes in states where Medicaid is more likely to cover the full cost of care have smaller racial and ethnic disparities in the availability of ASCUs than those in states where Medicaid payments cover the lowest proportion of care costs. These results are consistent with prior work that shows that racial and ethnic disparities in nursing home quality and staffing can often be associated with Medicaid payment policies.^23^ The costs in ASCUs are greater than for regular long-term care.^25^ In states with low Medicaid payment-to-cost ratios, admitting Medicaid residents could result in financial loss. Nursing homes may prefer to invest in ASCUs in areas where there are more private-pay residents, who are disproportionately White,^24^ thus contributing to the racial and ethnic disparity in the availability of ASCUs.

Nursing homes with higher percentages of either Black or Hispanic residents were less likely to have ASCUs. However, the association of higher Medicaid reimbursements with a reduction in those disparities was found only for nursing homes with a high proportion of Black residents. The MDS data in this study used only 1 question to residents to categorize both race and ethnicity, resulting in inaccuracies and undercounting of Hispanic residents. Following the new federal standards for collecting race and ethnicity data in 2024, the MDS now separates race from ethnicity, which should increase the accuracy of identifying Hispanic residents.^43^

Current federal regulations require states to ensure Medicaid payments are adequate to allow access for Medicaid residents equal to those with private pay, but they do not require that Medicaid payment rates cover the cost of care.^44^ The fact that the likelihood of having ASCUs was lower in nursing homes with higher proportions of Black residents who rely more on Medicaid suggests that some states may need to increase Medicaid payment rates to comply with this standard.

Our results are specific to the period before the COVID-19 pandemic. Research is needed to monitor the racial and ethnic disparities in ASCUs after the pandemic. Many nursing homes had chronic staffing shortages and increasing labor costs during the pandemic.^42,45^ In addition, many nursing homes were only able to operate during the pandemic due to public health emergency funds.^46^ As nursing homes stop receiving those funds, many facilities, especially those with a high proportion of Medicaid residents, may be forced to close more costly dementia units, further exacerbating disparities.

### Limitations

This study has some limitations. Beds in ASCUs were self-reported by nursing homes, and the criteria for ASCUs may differ among facilities, especially in states without specific regulations. We were unable to identify which individual residents were in an ASCU. We used federally mandated facility and resident data that had less than 1% missing data. Our analyses of Medicaid payment-to-cost ratios reflected the mean of all facilities in the state. Facility-level Medicaid payment-to-cost ratios could have a stronger association with ASCU availability in that facility. Medicaid base rates did not account for supplemental payments and other adjustments, which may underreport how much Medicaid pays certain facilities. Also, Medicaid costs were self-reported by nursing homes, and we did not account for payments to related parties.^47^ The mean Medicaid cost may not reflect the actual mean costs once all residents from all payer types (Medicaid, Medicare, private insurance, Veterans Affairs, and private pay) are included. Finally, our analyses did not include other state policies that might be associated with both the Medicaid payment-to-cost ratios and the availability of ASCUs, such as minimum nursing staff requirements.

## Conclusions

In this cohort study of US nursing homes, both the proportion of Black residents and the proportion of Hispanic residents were negatively associated with the availability of specialized dementia care units. But this negative association for the proportion of Black residents was not found in states with the highest Medicaid reimbursement rates. Racial disparities in specialized dementia care may be mitigated and even eliminated by more generous Medicaid payments.

## Notes

Supplement 1. **eFigure 1. **Consort Selection Diagram for Nursing Homes **eFigure 2. **Percentage of Nursing Homes With Alzheimer’s Special Care Units in Each State, 2019 **eFigure 3. **Relationship of the Percentage of Hispanic Residents in a Nursing Home and the Presence of an Alzheimer’s Special Care Unit, 2019 **eFigure 4.** Trend in Availability of Alzheimer’s Special Care Unit in Nursing Homes by Percentage of Black Residents, 2009-2019 **eFigure 5. **Trend in Availability of Alzheimer’s Special Care Unit in Nursing Homes by Percentage of Hispanic Residents, 2009-2019 **eFigure 6.** State Variation in Medicaid Payment-to-Cost Ratios, 2019 **eTable 1. **Race and Ethnicity Among Residents in 14,058 Nursing Homes in 2019 **eTable 2.** Definition of Variables Used in the Analysis **eTable 3.** Nursing Home Characteristics by Percentage of Black Residents, 2019 **eTable 4.** Nursing Home Characteristics by Percentage of Hispanic Residents, 2019 **eTable 5.** Generalized Estimating Equations Model Examining the Association of Percentage of Black Residents and Percentage of Hispanic Residents With Availability of Alzheimer’s Special Care Unit, 2009-2019 **eTable 6.** State Medicaid Reimbursement and Cost in 2019 **eTable 7.** Multivariable Logistic Regression Examining the Interaction of the Percentage of Black Residents With Medicaid Payment-to-Cost Ratios on the Availability of Alzheimer’s Special Care Unit, 2019 **eTable 8.** Disparities in the Availability of Alzheimer’s Special Care Unit by Medicaid Payment-to-Cost Ratios After Controlling for Percentage of State Medicaid Spending on Home and Community-Based Services, 2019 **eTable 9. **Multivariable Logistic Regression Examining the Interaction of the Percentage of Hispanic Residents With Medicaid Payment-to-Cost Ratios on the Availability of Alzheimer’s Special Care Unit, 2019 **eTable 10.** Differences in the Availability of Alzheimer’s Special Care Unit by Percentage of Hispanic Residents in States With Different Medicaid Payment-to- Cost Ratios, 2019

Supplement 2.
Data Sharing Statement
