# Supplementary material for: Medicaid Payments and Racial and Ethnic Disparities in Alzheimer Disease Special Care Units
Source: JAMA Netw Open. 2025 Aug 4;8(8):e2525057. doi: 10.1001/jamanetworkopen.2025.25057 (PMC12322793; doi:10.1001/jamanetworkopen.2025.25057)
Supplement: Supplement 1. — eFigure 1. Consort Selection Diagram for Nursing Homes eFigure 2. Percentage of Nursing Homes With Alzheimer’s Special Care Units in Each State, 2019 eFigure 3. Relationship of the Percentage of Hispanic Residents in a Nursing Home and the Presence of an Alzheimer’s Special Care Unit, 2019 eFigure 4. Trend in Availability of Alzheimer’s Special Care Unit in Nursing Homes by Percentage of Black Residents, 2009-2019 eFigure 5. Trend in Availability of Alzheimer’s Special Care Unit in Nursing Homes by Percentage of Hispanic Residents, 2009-2019 eFigure 6. State Variation in Medicaid Payment-to-Cost Ratios, 2019 eTable 1. Race and Ethnicity Among Residents in 14,058 Nursing Homes in 2019 eTable 2. Definition of Variables Used in the Analysis eTable 3. Nursing Home Characteristics by Percentage of Black Residents, 2019 eTable 4. Nursing Home Characteristics by Percentage of Hispanic Residents, 2019 eTable 5. Generalized Estimating Equations Model Examining the Association of Percentage of Black Residents and Percentage of Hispanic Residents With Availability of Alzheimer’s Special Care Unit, 2009-2019 eTable 6. State Medicaid Reimbursement and Cost in 2019 eTable 7. Multivariable Logistic Regression Examining the Interaction of the Percentage of Black Residents With Medicaid Payment-to-Cost Ratios on the Availability of Alzheimer’s Special Care Unit, 2019 eTable 8. Disparities in the Availability of Alzheimer’s Special Care Unit by Medicaid Payment-to-Cost Ratios After Controlling for Percentage of State Medicaid Spending on Home and Community-Based Services, 2019 eTable 9. Multivariable Logistic Regression Examining the Interaction of the Percentage of Hispanic Residents With Medicaid Payment-to-Cost Ratios on the Availability of Alzheimer’s Special Care Unit, 2019 eTable 10. Differences in the Availability of Alzheimer’s Special Care Unit by Percentage of Hispanic Residents in States With Different Medicaid Payment-to- Cost Ratios, 2019 [file jamanetwopen-e2525057-s001.pdf]

Supplementary Online Content

Xu H, Li S, Bowblis JR, Pappadis MR, Kuo YF, Goodwin JS. Medicaid payments and racial and ethnic disparities in Alzheimer disease special care units. *JAMA Netw Open.* 2025;8(8):e2525057. doi:10.1001/jamanetworkopen.2025.25057

eFigure 1. Consort Selection Diagram for Nursing Homes
eFigure 2. Percentage of Nursing Homes With Alzheimer's Special Care Units in Each State, 2019
eFigure 3. Relationship of the Percentage of Hispanic Residents in a Nursing Home and the Presence of an Alzheimer's Special Care Unit, 2019
eFigure 4. Trend in Availability of Alzheimer's Special Care Unit in Nursing Homes by Percentage of Black Residents, 2009-2019
eFigure 5. Trend in Availability of Alzheimer's Special Care Unit in Nursing Homes by Percentage of Hispanic Residents, 2009-2019
eFigure 6. State Variation in Medicaid Payment-to-Cost Ratios, 2019
eTable 1. Race and Ethnicity Among Residents in 14,058 Nursing Homes in 2019
eTable 2. Definition of Variables Used in the Analysis
eTable 3. Nursing Home Characteristics by Percentage of Black Residents, 2019
eTable 4. Nursing Home Characteristics by Percentage of Hispanic Residents, 2019
eTable 5. Generalized Estimating Equations Model Examining the Association of Percentage of Black Residents and Percentage of Hispanic Residents With Availability of Alzheimer's Special Care Unit, 2009-2019
eTable 6. State Medicaid Reimbursement and Cost in 2019
eTable 7. Multivariable Logistic Regression Examining the Interaction of the Percentage of Black Residents With Medicaid Payment-to-Cost Ratios on the Availability of Alzheimer's Special Care Unit, 2019
eTable 8. Disparities in the Availability of Alzheimer's Special Care Unit by Medicaid Payment-to-Cost Ratios After Controlling for Percentage of State Medicaid Spending on Home and Community-Based Services, 2019
eTable 9. Multivariable Logistic Regression Examining the Interaction of the Percentage of Hispanic Residents With Medicaid Payment-to-Cost Ratios on the Availability of Alzheimer's Special Care Unit, 2019
eTable 10. Differences in the Availability of Alzheimer's Special Care Unit by Percentage of Hispanic Residents in States With Different Medicaid Payment-to- Cost Ratios, 2019

This supplementary material has been provided by the authors to give readers additional information about their work.

© 2025 Xu H et al. *JAMA Network Open.*

eFigure 1. Consort Selection Diagram for Nursing Homes

Data on Medicaid payment-to-cost ratios in Alaska, Idaho, and New Hampshire were not available in the MACPAC report. The Certification and Survey Provider Enhanced Reporting data did not include nursing homes from the District of Columbia.

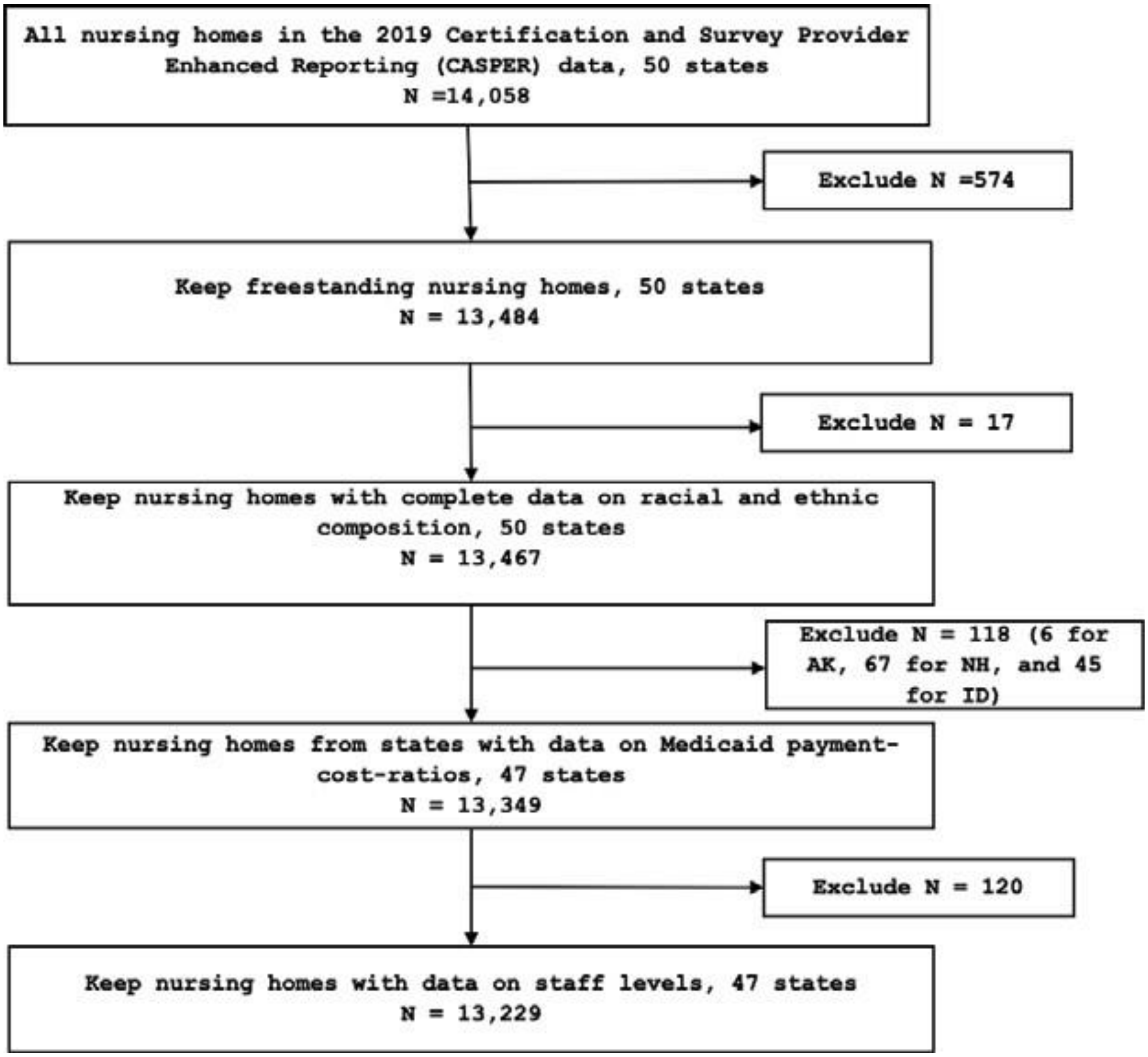

© 2025 Xu H et al. *JAMA Network Open.*

eFigure 2. Percentage of Nursing Homes With Alzheimer's Special Care Units in Each State, 2019

The sample included 14,058 nursing homes in 50 states from the 2019 Certification and Survey Provider Enhanced Reporting (CASPER) data. The percentage was calculated as the number of nursing homes with an Alzheimer's special care unit in a state divided by total number of nursing homes.

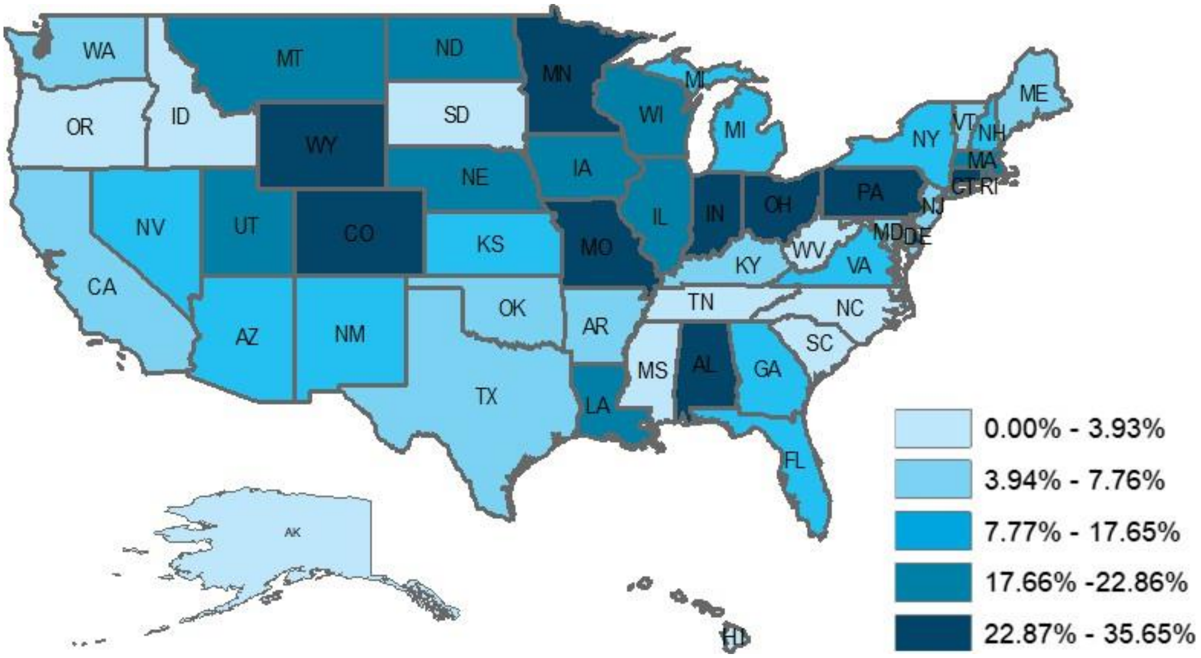

© 2025 Xu H et al. *JAMA Network Open.*

eFigure 3. Relationship of the Percentage of Hispanic Residents in a Nursing Home and the Presence of an Alzheimer's Special Care Unit, 2019

The sample included 13,179 nursing homes in 47 states from the 2019 Certification and Survey Provider Enhanced Reporting (CASPER) merged with Minimum Data Set 3.0 assessments. The percentage of Hispanic residents was calculated as number of Hispanic residents in 2019 in a facility divided by total number of residents. Nursing homes with the same percentage of Hispanic residents were grouped together. The number of nursing homes grouped at each percentile from 0% to 40% are ≥10. For percentiles >50% the number of facilities were usually <5. We therefore capped the X-axis at 50%. The dotted line represents a simple linear regression between percentage of Hispanic residents and Alzheimer's special care unit. The slope of the regression line was -0.14% (P=0.02).

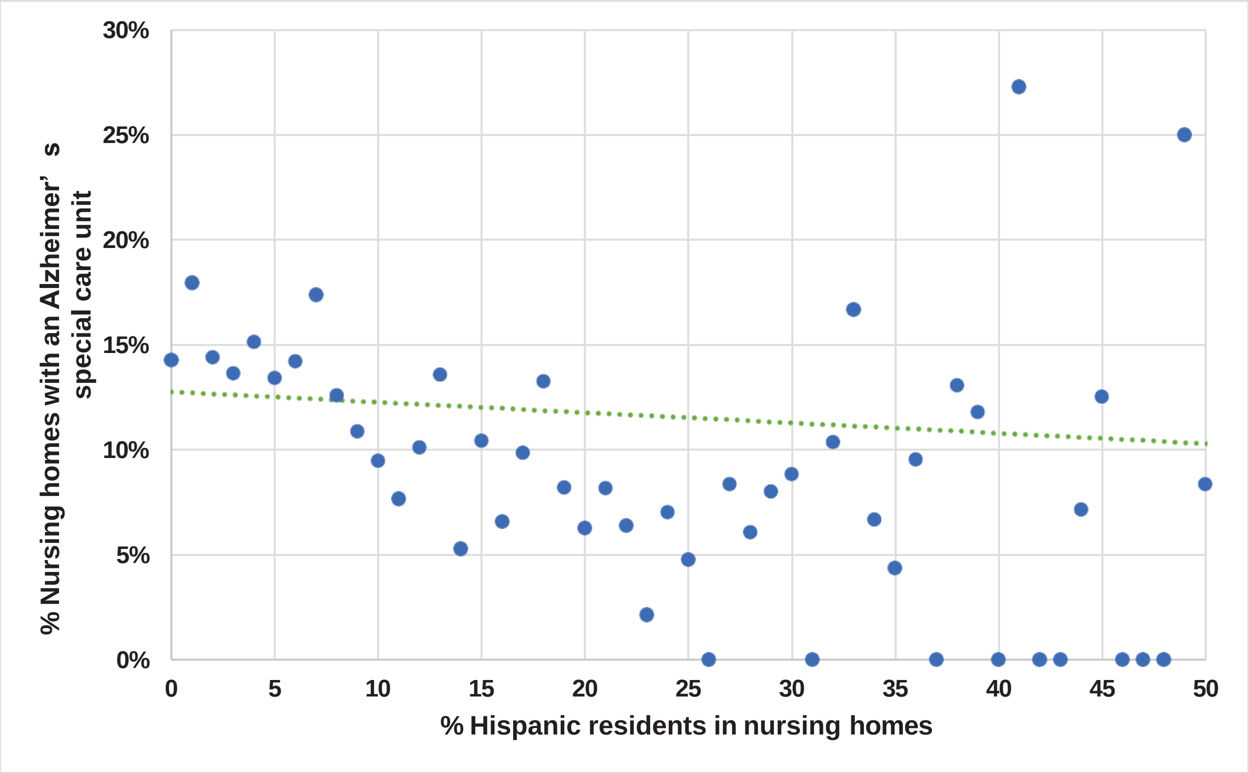

© 2025 Xu H et al. *JAMA Network Open.*

eFigure 4. Trend in Availability of Alzheimer’s Special Care Unit in Nursing Homes by Percentage of Black Residents, 2009-2019

The availability of Alzheimer’s special care unit was obtained from the Certification and Survey Provider Enhanced Reporting (CASPER) data. The CASPER surveys occur every 9 to 15 months so not every nursing home had data in each year. The number of nursing homes ranged from 14,369 to 15,186 each year over the period of 2009-2019. Nursing homes were categorized into 4 quartiles for each year based on percentage of Black residents in a facility. The group that a facility was assigned could change over time if the percentage of Black residents changed.

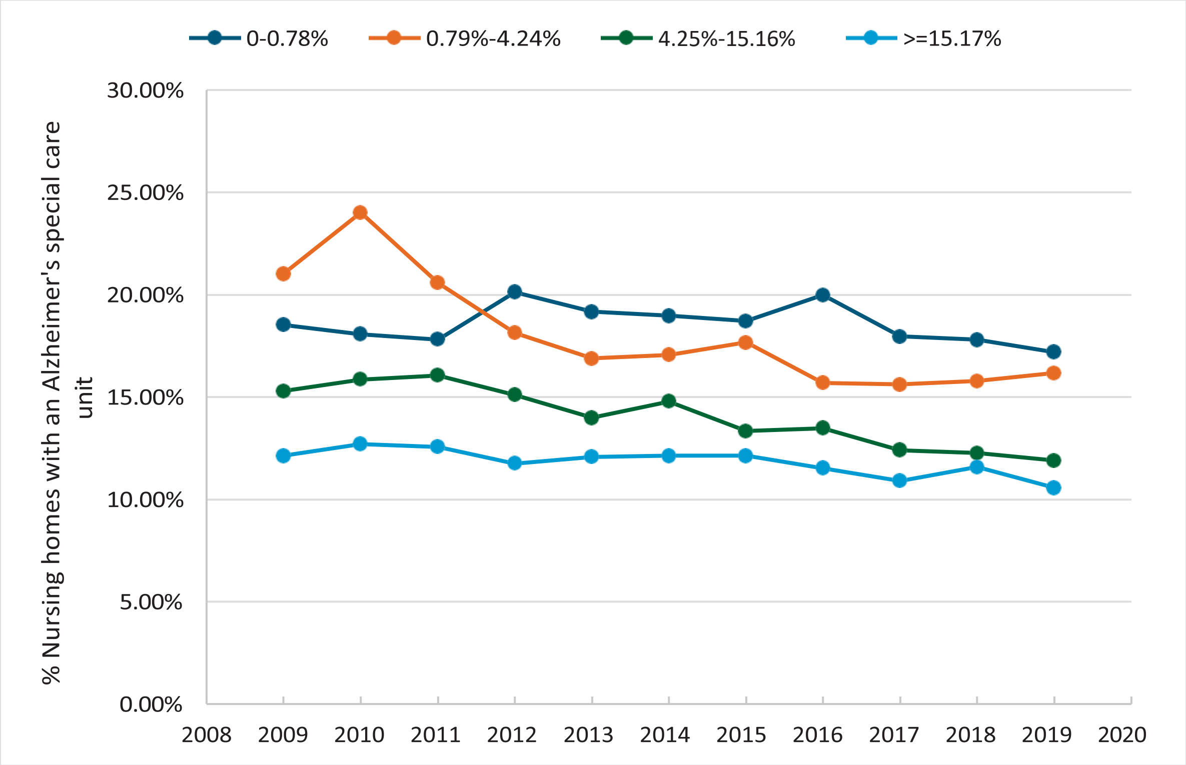

© 2025 Xu H et al. *JAMA Network Open.*

eFigure 5. Trend in Availability of Alzheimer's Special Care Unit in Nursing Homes by Percentage of Hispanic Residents, 2009-2019

The availability of Alzheimer's special care unit was obtained from the Certification and Survey Provider Enhanced Reporting (CASPER) data. The CASPER surveys occur every 9 to 15 months so not every nursing home had data in each year. The number of nursing homes ranged from 14,369 to 15,186 each year over the period of 2009-2019. Nursing homes were categorized into 4 quartiles for each year based on percentage of Hispanic residents in a facility. The group that a facility was assigned could change over time if the percentage of Hispanic residents changed.

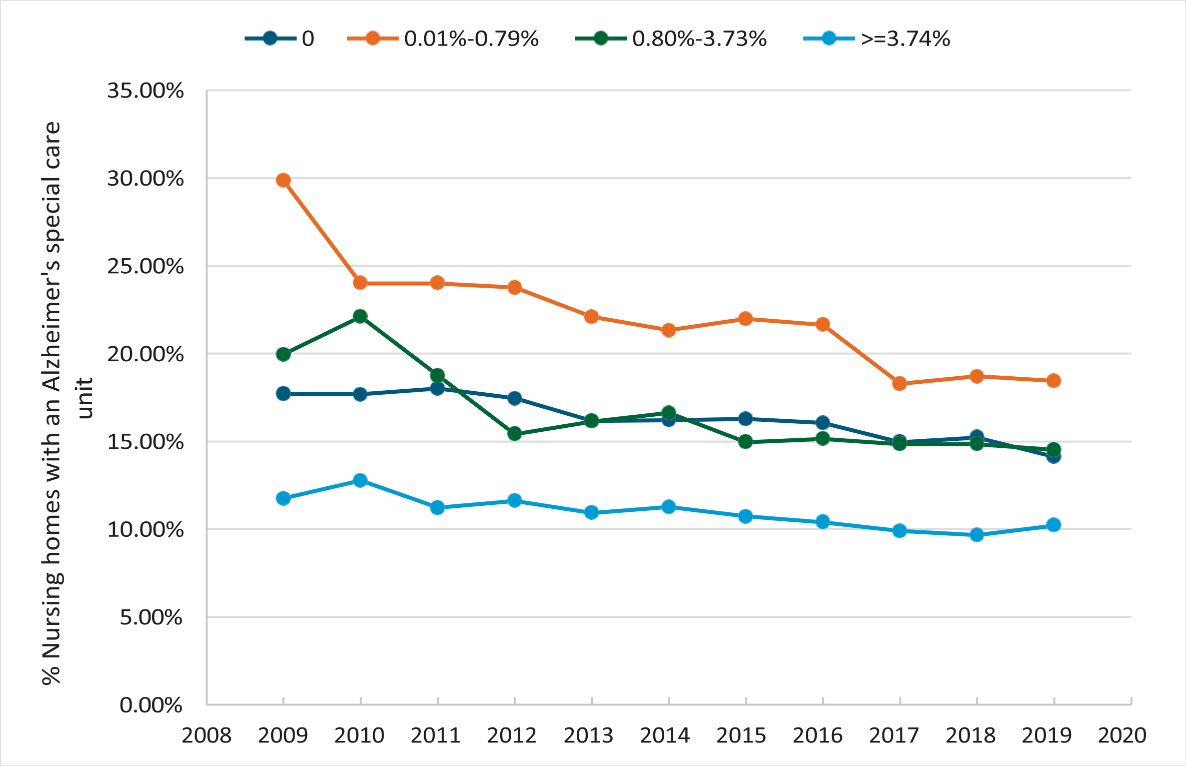

© 2025 Xu H et al. *JAMA Network Open.*

eFigure 6. State Variation in Medicaid Payment-to-Cost Ratios, 2019

Medicaid payment-cost ratios were calculated as the average Medicaid payment rate divided by average Medicaid nursing home cost for each state. Data in Alaska, Idaho, and New Hampshire were not available in the MACPAC report. States were grouped based on the quartiles of Medicaid payment-to-cost ratios.

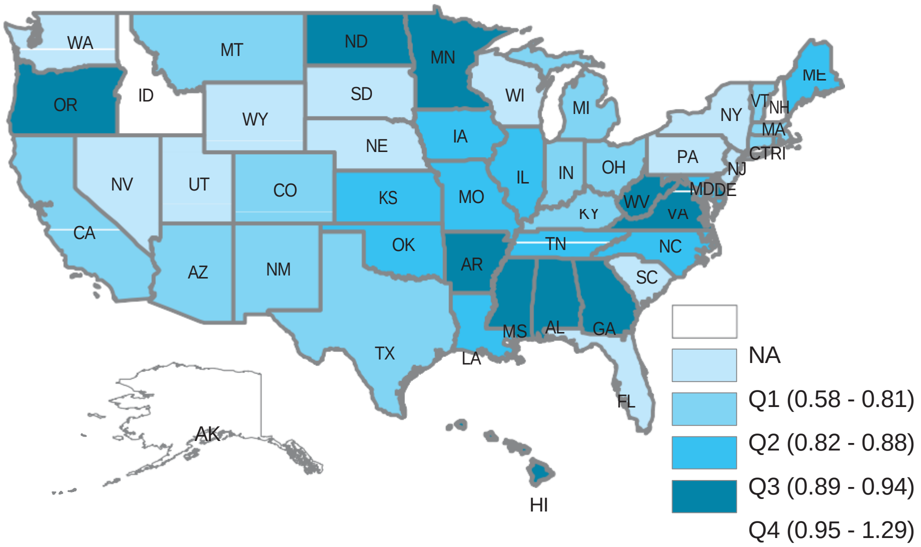

© 2025 Xu H et al. *JAMA Network Open.*

eTable 1. Race and Ethnicity Among Residents in 14,058 Nursing Homes in 2019

Race and ethnicity	All residents (N=3,817,187)
American Indian or Alaska Native, n(%)	15,692 (0.41%)
Asian, n(%)	74,417 (1.95%)
Black or African American, n(%)	471,922 (12.36%)
Hispanic or Latino, n(%)	191,810 (5.02%)
Native Hawaiian or Other Pacific Islander, n(%)	6,318 (0.17%)
White, n(%)	2,861,493 (74.66%)

Note: Race and ethnicity information were self-reported by residents, collected in the Minimum Data Set 3.0. The percentage was calculated for all nursing home residents who had any MDS assessment in a facility in 2019. Because there was only one question for race and ethnicity, we cannot identify both the race and ethnicity of residents.

© 2025 Xu H et al. *JAMA Network Open.*

eTable 2. Definition of Variables Used in the Analysis

Variables	Definition
**Alzheimer's special care units**	At least one Alzheimer's special care unit bed in a facility (CASPER, F16)
**Percentage of Black residents in a facility**	The percentage of Black residents among all residents who have at least one Minimum Data Set assessment (MDS A1000), and stratified by quartiles
**Percentage of Hispanic residents in a facility**	The percentage of Hispanic residents among all residents who have at least one Minimum Data Set assessment (MDS A1000), and stratified by quartiles
**Medicaid payment-to-cost ratio**	The ratio of average state Medicaid payment to nursing home divided by the average nursing home cost for Medicaid residents in a state (MACPAC), and stratified by quartiles
**Ownership status**	Whether a nursing home is for-profit vs. non-profit or government (CASPER)
**Chain affiliation**	Part of a multi-facility chain (CASPER)
**Location**	Located in urban or rural area using zip code linked to the Rural-Urban Commuting Area codes (CASPER, zip code)
**Bed size**	Total number of beds in a nursing home (CASPER)
**Percentage of Medicaid residents**	The percentage of residents whose primary payer is Medicaid as of the day of survey (CASPER, F75)
**Percentage of Medicare residents**	The percentage of residents whose primary payer is Medicare as of the day of survey (CASPER, F76)
**Percentage of residents with dementia**	The percentage of residents with a diagnosis of dementia as of the day of survey (CASPER, F111)
**Percentage of residents with depression**	The percentage of residents with a diagnosis of depression as of the day of survey (CASPER, F109)
**Percentage of residents with serious mental illness**	The percentage of residents with a diagnosis of schizophrenia, schizoaffective disorder, schizophreniform disorder, delusional disorder, psychotic mood disorders, or anxiety disorders as of the day of survey (CASPER, F110)
**Total staff hours per resident day**	Total staff hours from registered nurses, licensed practical nurses, and certified nurse aides divided by total number of resident days (Payroll-based Journal), and stratified by quartiles
**Percentage of Medicaid long term services and supports spending on Home and Community-Based Services (HCBS)**	State Medicaid spending on HCBS as a percentage of all Medicaid spending on long term services and supports (HCBS and institutional care)

Notes: CAPSER, Certification and Survey Provider Enhanced Reporting; MACPAC, Medicaid and CHIP Payment and Access Commission. The item numbers are from the Minimum Data Set 3.0 assessments for residents, and CMS Form 671 and Form 672 for facilities.

© 2025 Xu H et al. *JAMA Network Open.*

eTable 3. Nursing Home Characteristics by Percentage of Black Residents, 2019

Variables	All nursing homes	Percentage of Black residents				
		Q1 (0-0.78%)	Q2 (0.79%-4.24%)	Q3 (4.25%-15.16%)	Q4 (≥15.17%)	P value[a]
	(N=13,229)	(N=3,307)	(N=3,307)	(N=3,308)	(N=3,307)	
	Mean (SD)	Mean (SD)	Mean (SD)	Mean (SD)	Mean (SD)	
**Alzheimer's special care unit, n(%)**	1,864 (14.09%)	581 (17.57%)	537 (16.24%)	395 (11.94%)	351 (10.61%)	<0.001
**For-profit, n(%)**	9,561 (72.27%)	1753 (53.01%)	2380 (71.97%)	2635 (79.66%)	2793 (84.46%)	<0.001
**Part of a chain, n(%)**	7,775 (58.77%)	1755 (53.07%)	2031 (61.42%)	2026 (61.25%)	1963 (59.36%)	<0.001
**Urban, n(%)**	9,393 (71.00%)	1501 (45.39%)	2328 (70.40%)	2763 (83.52%)	2801 (84.70%)	<0.001
**Total staff hours per resident day**	3.82 (0.87)	3.99 (0.90)	3.88 (0.85)	3.78 (0.87)	3.64 (0.82)	<0.001
**Total number of beds**	109.12 (57.36)	83.69 (44.35)	105.64 (52.21)	117.77 (58.86)	129.37 (62.16)	<0.001
**% Medicaid residents (0-100)**	59.76 (23.68)	52.75 (22.70)	55.36 (24.66)	61.29 (23.41)	69.62 (20.09)	<0.001
**% Medicare residents (0-100)**	12.80 (12.76)	11.79 (11.89)	14.62 (14.69)	13.60 (13.21)	11.19 (10.60)	<0.001
**% Residents with dementia (0-100)**	44.37 (17.21)	47.72 (16.96)	45.05 (17.06)	43.43 (17.57)	41.30 (16.59)	<0.001
**% Residents with depression (0-100)**	36.42 (23.45)	41.66 (23.37)	38.09 (23.34)	35.28 (22.96)	30.63 (22.75)	<0.001
**% Residents with serious mental illness (0-100)**	33.71 (18.45)	30.41 (16.66)	32.27 (17.76)	35.70 (19.55)	36.45 (19.02)	<0.001

Note: [a] P values comparing nursing home characteristics by racial and ethnic composition were from chi-square statistics for categorical variables and ANOVA F statistics for continuous variables. Facility characteristics significantly differed by the percentage of Black residents in a facility. For example, nursing homes with higher proportions of Black residents were more likely to be for-profit, from urban areas, have lower staff levels, and rely on Medicaid payments.

© 2025 Xu H et al. *JAMA Network Open.*

eTable 4. Nursing Home Characteristics by Percentage of Hispanic Residents, 2019

**Variables**	**All nursing homes**	**Percentage of Hispanic residents**				
		**Q1 (0%)**	**Q2 (0.01%-0.79%)**	**Q3 (0.80%-3.73%)**	**Q4 (≥3.74%)**	**P value**[a]
	**(N=13,229)**	**(N=4,346)**	**(N=2,259)**	**(N=3,316)**	**(N=3,308)**	
	**Mean (SD)**	**Mean (SD)**	**Mean (SD)**	**Mean (SD)**	**Mean (SD)**	
**Alzheimer's special care unit, n(%)**	1,864 (14.09%)	620 (14.27%)	419 (18.55%)	486 (14.66%)	339 (10.25%)	<0.001
**For-profit, n(%)**	9,561 (72.27%)	2,730 (62.82%)	1,555 *68.84%)	2,483 (74.88%)	2,793 (84.43%)	<0.001
**Part of a chain, n(%)**	7,775 (58.77%)	2,401 (55.25%)	1,440 (63.75%)	2,088 (62.97%)	1,846 (55.80%)	<0.001
**Urban, n(%)**	9,393 (71.00%)	2,163 (49.77%)	1,734 (76.76%)	2,560 (77.20%)	2,936 (88.75%)	<0.001
**Total staff hours per resident day**	3.82 (0.87)	3.86 (0.90)	3.91 (0.78)	3.81 (0.89)	3.72 (0.86)	<0.001
**Total number of beds**	109.12 (57.36)	87.03 (42.12)	120.51 (52.77)	114.56 (59.44)	124.91 (66.17)	<0.001
**% Medicaid residents (0-100)**	59.76 (23.68)	58.60 (23.52)	54.96 (23.90)	59.60 (23.90)	64.70 (22.60)	<0.001
**% Medicare residents (0-100)**	12.80 (12.76)	10.99 (11.07)	15.60 (14.22)	13.80 (14.05)	12.27 (11.96)	<0.001
**% Residents with dementia (0-100)**	44.37 (17.21)	47.21 (16.37)	42.95 (16.18)	42.67 (16.98)	43.33 (18.67)	<0.001
**% Residents with depression (0-100)**	36.42 (23.45)	40.19 (24.16)	37.23 (22.31)	37.15 (22.90)	30.17 (22.57)	<0.001
**% Residents with serious mental illness (0-100)**	33.71 (18.45)	35.01 (18.29)	31.78 (16.28)	33.59 (18.59)	33.43 (19.73)	<0.001

Note: [a] P values comparing nursing home characteristics by racial and ethnic composition were from chi-square statistics for categorical variables and ANOVA F statistics for continuous variables. Facility characteristics significantly differed by the percentage of Hispanic residents in a facility. For example, nursing homes with higher proportions of Hispanic residents were more likely to be for-profit, from urban areas, have lower staff levels, and rely on Medicaid payments.

© 2025 Xu H et al. *JAMA Network Open.*

eTable 5. Generalized Estimating Equations Model Examining the Association of Percentage of Black Residents and Percentage of Hispanic Residents With Availability of Alzheimer's Special Care Unit, 2009-2019

**Facility characteristics**	**Adjusted Odds Ratio (95% CI)**
**Percentage of Black residents, each 10-unit increase**	0.90 (0.89, 0.92)
**Percentage of Hispanic residents, each 10-unit increase**	0.85 (0.82, 0.88)
**Profit status**	
For-profit	Ref
Non-profit or government	1.39 (1.31, 1.48)
**Part of a chain**	
No	Ref
Yes	1.10 (1.05, 1.15)
**Urban**	
No	Ref
Yes	0.71 (0.65, 0.77)
**Bed size, 10-bed increase**	1.09 (1.08, 1.10)
**Percentage of Medicaid residents, 10-unit increase**	0.99 (0.98, 1.01)
**Percentage of Medicare residents, 10-unit increase**	0.97 (0.95, 0.98)
**Percentage of residents with dementia, 10-unit increase**	1.05 (1.04, 1.06)
**Percentage of residents with depression, 10-unit increase**	1.01 (1.01, 1.02)
**Percentage of residents with serious mental illness, 10-unit increase**	0.99 (0.99, 1.01)
**Year**	
2009	Ref
2010	1.00 (0.99, 1.01)
2011	0.99 (0.98, 1.01)
2012	0.96 (0.94, 0.98)
2013	0.91 (0.88, 0.94)
2014	0.93 (0.90, 0.97)
2015	0.92 (0.88, 0.96)
2016	0.92 (0.88, 0.96)
2017	0.85 (0.81, 0.89)
2018	0.85 (0.81, 0.89)
2019	0.83 (0.79, 0.88)

Notes: The sample included we included 162,813 nursing home facility-year observations over 2009-2019, ranging from 14,369 to 15,186 facilities per year. Generalized estimating equations (GEE) with logit link and an autoregressive (AR1) working correlation was conducted to account for the nested nature of data (multiple observations for each facility). The percentage of Black residents and percentage of Hispanic residents were included as continuous variables. We did not include staff hours per resident day because the Payroll-Based Journal data were not available before 2017.

© 2025 Xu H et al. *JAMA Network Open.*

eTable 6. State Medicaid Reimbursement and Cost in 2019

Variable	Number of states with available data [a]	Mean of all states with available data	Std. Dev.	Min	Max
**State average Medicaid base payment rates to nursing homes[b]**	47	$212.1	$53.8	$125.5	$375.8
**State average Medicaid nursing home stay costs[c]**	47	$244.6	$38.5	$169.7	$318.8
**Medicaid payment-cost ratios[d]**	47	0.87	0.13	0.58	1.29

Notes: [a] Data in Alaska, Idaho, and New Hampshire were not available in the Medicaid and CHIP Payment and Access Commission report. MLTSS programs in those states accounted for a large share of overall Medicaid LTSS spending.
[b] The calculation only included Medicaid base payment rates and excluded Medicaid supplemental payments to nursing homes because complete data on supplemental payments to nursing facilities are not yet available at the facility level.
[c] Average costs were adjusted for resident acuity.
[d] Medicaid payment-cost ratios were calculated as the average Medicaid base payment rate divided by average Medicaid nursing home cost for each state.

© 2025 Xu H et al. *JAMA Network Open.*

eTable 7. Multivariable Logistic Regression Examining the Interaction of the Percentage of Black Residents With Medicaid Payment-to-Cost Ratios on the Availability of Alzheimer's Special Care Unit, 2019

**Facility characteristics**	**Coefficient (standard error)**	**P value**
**Percentage of Black residents**		
Q1 (0-0.78%)	Ref	
Q2 (0.79%-4.24%)	-0.19 (0.15)	0.19
Q3 (4.25%-15.16%)	-0.58 (0.16)	<0.01
Q4 (≥15.17%)	-1.28 (0.19)	<0.01
**Medicaid payment-to-cost ratio**		
Q1 (0.58-0.81)	Ref	
Q2-Q3 (0.82-0.94)	0.06 (0.11)	0.57
Q4 (0.94-1.29)	-0.35 (0.17)	0.04
**Interaction of percentage of Black residents and Medicaid payment-to-cost ratio for the availability of availability of Alzheimer's special care units**		<0.01
Medicaid ratio Q2-Q3 * Percentage of Black residents Q2	0.04 (0.17)	0.81
Medicaid ratio Q2-Q3 * Percentage of Black residents Q3	0.002 (0.18)	0.99
Medicaid ratio Q2-Q3 * Percentage of Black residents Q4	0.69 (0.20)	<0.01
Medicaid ratio Q4 * Percentage of Black residents Q2	0.54 (0.27)	0.04
Medicaid ratio Q4 * Percentage of Black residents Q3	0.52 (0.29)	0.07
Medicaid ratio Q4 * Percentage of Black residents Q4	1.23 (0.27)	<0.01

Note: The outcome is the availability of availability of Alzheimer's special care unit. The model controlled for total staff hours per resident day, profit status, ownership, chain affiliation, location, bed size, percentage of Medicaid residents, percentage of Medicare residents, percentage of residents with dementia, percentage of residents with depression, and percentage of residents with serious mental illness. When treating both the percentage of Black residents in a facility and Medicaid payment-to-cost-ratios as continuous variables, the interaction term remained significant (Chi-square statistic= 30.07, P<0.01).

© 2025 Xu H et al. *JAMA Network Open.*

eTable 8. Disparities in the Availability of Alzheimer's Special Care Unit by Medicaid Payment-to-Cost Ratios After Controlling for Percentage of State Medicaid Spending on Home and Community-Based Services, 2019

	**State Medicaid payment-to-cost ratios [b]**		
	**Q1(0.58-0.81)**	**Q2-Q3(0.82-0.94)**	**Q4(0.94-1.29)**
	Odds Ratio (95% CI)	Odds Ratio (95% CI)	Odds Ratio (95% CI)
**Percentage of Black residents**			
Q1 (0-0.78%)	Ref	Ref	Ref
Q2 (0.79%-4.24%)	0.85 (0.62, 1.17)	0.85 (0.71, 1.01)	1.80 (1.09, 2.97)
Q3 (4.25%-15.16%)	0.63 (0.43, 0.93)	0.57 (0.46, 0.69)	1.24 (0.69, 2.23)
Q4 (≥15.17%)	0.33 (0.22, 0.52)	0.56 (0.44, 0.70)	1.67 (0.95, 2.94)
**Number of nursing homes**	3,060	8,589	1,580
**Number of states [a]**	12	24	11

Notes: [a] Three separate multivariable logistic regressions with robust standard errors were conducted in states with Medicaid payment-to-cost ratios in Q1, Q2-Q3, and Q4. The quartiles of Medicaid ratios were based on the number of states. Models controlled for the same facility characteristics as in Table 1.

[b] Nursing homes in three states and the District of Columbia were excluded from the analysis. Data in Alaska, Idaho, and New Hampshire were not available in the MACPAC report. The Certification and Survey Provider Enhanced Reporting data did not include nursing homes from the District of Columbia. Data on percentage of state Medicaid spending on HCBS were unavailable in California, Delaware, Illinois, and Virgina because those states did not submit Managed Long-Term Services and Support expenditure data for 2019, and because their Managed Long-Term Services and Support programs account for a large share of overall Medicaid Long-Term Services and Support spending. We included percentage of state Medicaid spending on Home and Community-Based Services as a categorical variable: states with no data, Q1 (HCBS ≤46.9%), Q2 (47.0%-54.2%), Q3 (54.3%-64.1%), and Q4 (≥64.2%).

© 2025 Xu H et al. *JAMA Network Open.*

eTable 9. Multivariable Logistic Regression Examining the Interaction of the Percentage of Hispanic Residents With Medicaid Payment-to-Cost Ratios on the Availability of Alzheimer's Special Care Unit, 2019

**Facility characteristics**	**Coefficient (standard error)**	**P value**
**Percentage of Hispanic residents**		
Q1 (0%)	Ref	
Q2 (0.01%-0.79%)	0.39(0.16)	0.02
Q3 (0.80%-3.73%)	0.10 (0.15)	0.05
Q4 (≥3.74%)	-0.43 (0.17)	0.01
**Medicaid payment-to-cost ratio**		
Q1 (0.58-0.81)	Ref	
Q2-Q3 (0.82-0.94)	0.19 (0.12)	0.11
Q4 (0.94-1.29)	-0.02 (0.15)	0.88
**Interaction of percentage of Hispanic residents and Medicaid payment-to-cost ratio for the availability of availability of Alzheimer's special care units**		0.37
Medicaid ratio Q2-Q3 * Percentage of Hispanic residents Q2	-0.05 (0.19)	0.77
Medicaid ratio Q2-Q3 * Percentage of Hispanic residents Q3	-0.02 (0.17)	0.89
Medicaid ratio Q2-Q3 * Percentage of Hispanic residents Q4	-0.01 (0.19)	0.95
Medicaid ratio Q4 * Percentage of Hispanic residents Q2	-0.17 (0.25)	0.50
Medicaid ratio Q4 * Percentage of Hispanic residents Q3	-0.04 (0.25)	0.88
Medicaid ratio Q4 * Percentage of Hispanic residents Q4	1.27 (0.56)	0.02

Note: The outcome is the availability of availability of Alzheimer's special care unit. The model controlled for total staff hours per resident day, profit status, ownership, chain affiliation, location, bed size, percentage of Medicaid residents, percentage of Medicare residents, percentage of residents with dementia, percentage of residents with depression, and percentage of residents with serious mental illness. The overall interaction of percentage of Hispanic residents and Medicaid ratios was not significant, but the interaction of Medicaid ratio in Q4 and percentage of Hispanic residents in Q4 was significant. This could be due to random chance, because the standard error for this interaction was much larger than the remaining estimates.

© 2025 Xu H et al. *JAMA Network Open.*

eTable 10. Differences in the Availability of Alzheimer's Special Care Unit by Percentage of Hispanic Residents in States With Different Medicaid Payment-to- Cost Ratios, 2019

	**State Medicaid payment-to-cost ratios [a]**		
	**Q1(0.58-0.81)**	**Q2-Q3(0.82-0.94)**	**Q4(0.94-1.29)**
	Odds Ratio (95% CI)	Odds Ratio (95% CI)	Odds Ratio (95% CI)
**Percentage of Hispanic residents**			
Q1 (0%)	Ref	Ref	Ref
Q2 (0.01%-0.79%)	1.85 (1.31, 2.61)	1.44 (1.18, 1.75)	0.92 (0.61, 1.38)
Q3 (0.80%-3.73%)	1.45 (1.04, 2.03)	1.11 (0.93, 1.33)	0.85 (0.55, 1.30)
Q4 (≥3.74%)	1.20 (0.80, 1.80)	0.67 (0.55, 0.81)	2.00 (0.67, 5.95)
**Number of nursing homes**	3,060	8,589	1,580
**Number of states [b]**	12	24	11

Notes: [a] Three separate multivariable logistic regressions with robust standard errors were conducted in states with Medicaid payment-to-cost ratios in Q1, Q2-Q3, and Q4. The quartiles of Medicaid ratios were based on the number of states. Models controlled for the same facility characteristics as in Table 1. The interaction of percentage of Hispanic residents and Medicaid ratios was not significant (P=0.37). The findings are consistent with results from one logistic regression with the interaction term of the percentage of Hispanic residents with Medicaid ratios.
[b] Nursing homes in three states and the District of Columbia were excluded from the analysis. Data in Alaska, Idaho, and New Hampshire were not available in the MACPAC report. The Certification and Survey Provider Enhanced Reporting data did not include nursing homes from the District of Columbia.

© 2025 Xu H et al. *JAMA Network Open.*
