# Supplementary material for: Medicaid Payments and Racial and Ethnic Disparities in Alzheimer Disease Special Care Units
Source: JAMA Netw Open. 2025 Aug 4;8(8):e2525057. doi: 10.1001/jamanetworkopen.2025.25057 (PMC12322793; doi:10.1001/jamanetworkopen.2025.25057)
Supplement: Supplement 2. — Data Sharing Statement [file jamanetwopen-e2525057-s002.pdf]

Data Sharing Statement

Xu. Medicaid Payments and Racial and Ethnic Disparities in Alzheimer Disease Special Care Units. *JAMA Netw Open*. Published August 04, 2025.
doi:10.1001/jamanetworkopen.2025.25057

Data
Data available: No

Additional Information
Explanation for why data not available: We used public data from CMS and MACPAC.
